# Signatures of Polar Metal Phase in the Quasi-2D Electron System in PLD-Grown Amorphous-Epitaxial Oxide Heterostructures

**DOI:** 10.1021/acs.nanolett.5c03409

**Published:** 2025-08-31

**Authors:** Alessia Sambri, Yu Chen, Federico Mazzola, Emiliano Di Gennaro, Andrea Rubano, Martando Rath, Domenico Paparo, Marco Caputo, Alla Chikina, Deepak Kumar, Vladimir N. Strocov, Marco Salluzzo, Fabio Miletto Granozio

**Affiliations:** † CNR-SPIN, Institute for SuPerconductors, INnovative materials and devices, Unit of Naples, Complesso Universitario di Monte Sant’Angelo, via Cinthia, 80126 Napoli, Italy; ‡ Dipartimento di Fisica “E. Pancini”, Università degli Studi di Napoli “Federico II”, Complesso Universitario di Monte Sant’Angelo, via Cinthia, 80126 Napoli, Italy; § CNR-ISASI, Institute of Applied Sciences and Intelligent Systems “E. Caianiello”, 80078 Pozzuoli, NA Italy; ∥ Swiss Light Source, Paul Scherrer Institut, CH-5232 Villigen PSI, Switzerland

**Keywords:** Polar metals, two-dimensional electron gas (2DEG), BaTiO_3_-based oxide heterostructures, Second Harmonic Generation (SHG), Synchrotron-based spectroscopy

## Abstract

For years, itinerant charge carriers in ferroelectric insulators were believed to completely quench ferroelectricity. Recent breakthroughs, however, demonstrated the existence of a novel class of quasi-two-dimensional polar metals with promising applications in nonvolatile electronics and spintronics. Here, by combining temperature-dependent magnetotransport measurements, optical second harmonic generation (SHG), resonant photoemission spectroscopy (ResPES), and X-ray absorption spectroscopy (XAS), we report on the properties of a BaTiO_3_-based oxide heterostructure, sustaining a persistent polar displacement in the BaTiO_3_ layer while supporting a two-dimensional electron gas. This suggests that the oxide heterostructure may operate as a polar metal system, paving the way for new developments in oxide-based electronics.

## Graphical abstract

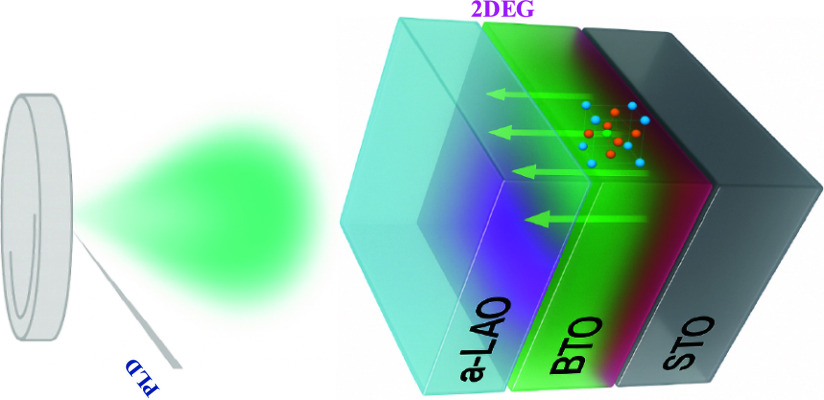

The concept of ferroelectric-like transitions—marked by the onset of polar order—in metals has intrigued the scientific community for over 50 years.^1^ Despite early theoretical predictions,^2^ experimental validation of true polar metals has only recently gained momentum, through an increasing number of reports on materials possibly combining polar symmetry with metallic conductivity, as reviewed in ref 3. This renewed interest is fueled by the promise of integrating diverse functionalities into a single material system. The seminal observation of a ferroelectric-like transition in LiOsO_3_ ^4^ and WTe_2_,^5^ also showing electrically switchable ferroelectricity, were landmark achievements. More recently, advances in epitaxial growth techniques enabled the synthesis of oxide heterostructures combining interfacial conductivity with ferroelectricity and magnetism.^6–8^ Such systems are two-dimensional (2D) ferroelectric metals, showing nonvolatile switching of magneto-transport properties induced by the reversal of FE polarization.

In this work, we present results on a novel kind of oxide heterostructure hosting a 2D electron gas (2DEG), realized by embedding crystalline BaTiO_3_ (BTO) films between an amorphous LaAlO_3_ (a-LAO) film and a SrTiO_3_ (STO) single crystal. Through a comprehensive multimodal approach—including temperature-dependent electrical measurements, optical second harmonic generation (SHG), resonant photoemission electron spectroscopy (ResPES), and X-ray absorption spectroscopy (XAS)—we demonstrate that the a-LAO/BTO/STO system represents a novel example of polar 2D metal. The heterostructure exhibits a 2DEG confined within the BTO layer and the first interfacial STO unit cells (uc) and is characterized by significant Ti-polar displacements, similar to those found in bulk ferroelectric BTO. Our results suggest that interactions between BTO and STO may stabilize this phase, providing a new perspective for engineering polar metals in oxides.

The LAO/STO interface is renowned for the concomitant occurrence of quite extraordinary electric field-tunable properties, as high carrier mobility, quantum transport phenomena, including quantum Hall effect, superconductivity, and magnetism.^9–13^ BTO, a prototypical ferroelectric perovskite, undergoes a phase transition from paraelectric to ferroelectric characterized by Ti^4+^ ion displacements. Recent research has shown that BTO single crystals, when doped with oxygen vacancies, remain ferroelectric even at high carrier concentrations, while still exhibiting a metallic behavior.^14–16^ Beside the aforementioned case of BaTiO_3−δ_ single crystals, a polar metallic phase has been reported also for (Ba_0.97_Sr_0.03_)_0.98_La_0.02_TiO_3_ single crystal,^17^ trilayer BTO­(10uc)/STO­(3uc)/LaTiO_3_(3uc) superlattices grown on Nb:STO,^18^ epitaxial LAO­(15uc)/Ba_0.5_Sr_0.5_TiO_3_(10uc)/STO heterostructure^19^ and Ba_0.5_La_0.5_TiO_3_/STO.^20^ Building on these insights, we designed and fabricated a series of BTO­(*n*)/STO heterostructures (where 3 ≤ *n* ≤ 20 uc), capped with an amorphous LAO film to explore the potential of creating a novel polar metal through the integration of these materials. Among the aforementioned examples, the present heterointerface represents the first polar metal reported in a mixed amorphous–crystalline heterostructure. Such a simpler system, in terms of fabrication complexity, overcomes at the same time the statement relative to the need of weakening the BTO ferroelectricity through Sr or La doping to avoid a semiconducting behavior at low temperature, showing the coexistence of a metallic behavior and a polar displacement in BTO from a minimum thickness of 1.2 nm up to (at least) 5.6 nm, as discussed in the following.

The heterostructures were deposited by pulsed laser deposition (PLD) in a multitarget ultrahigh vacuum (UHV) chamber, with calibration of BTO thickness via RHEED intensity oscillations and a-LAO thickness determined through atomic force microscopy (AFM). Figure 1a shows RHEED specular spot intensity oscillations for 12 uc BTO and the corresponding RHEED final pattern. Figure 1b shows the temperature-dependent sheet resistance (*R*–*T*) of a set of a-LAO/BTO/STO samples with different BTO thickness and fixed a-LAO thickness. Our temperature-dependent sheet resistance measurements reveal a transition from metallic to insulating behavior as BTO thickness increases, with metallic properties observed up to 14 uc and insulating behavior beyond 20 uc. Control experiments on BTO/STO samples confirm that BTO alone remains insulating. This highlights the necessity of the a-LAO layer for conductivity, with a minimum threshold thickness of 2.5 nm, below which the whole system is insulating. Similar behavior has been reported in ref 19 in the case of epitaxial LaAlO_3_(15uc)/Ba_0.5_Sr_0.5_TiO_3_(10uc)/SrTiO_3_ heterostructures.

**Figure 1:**
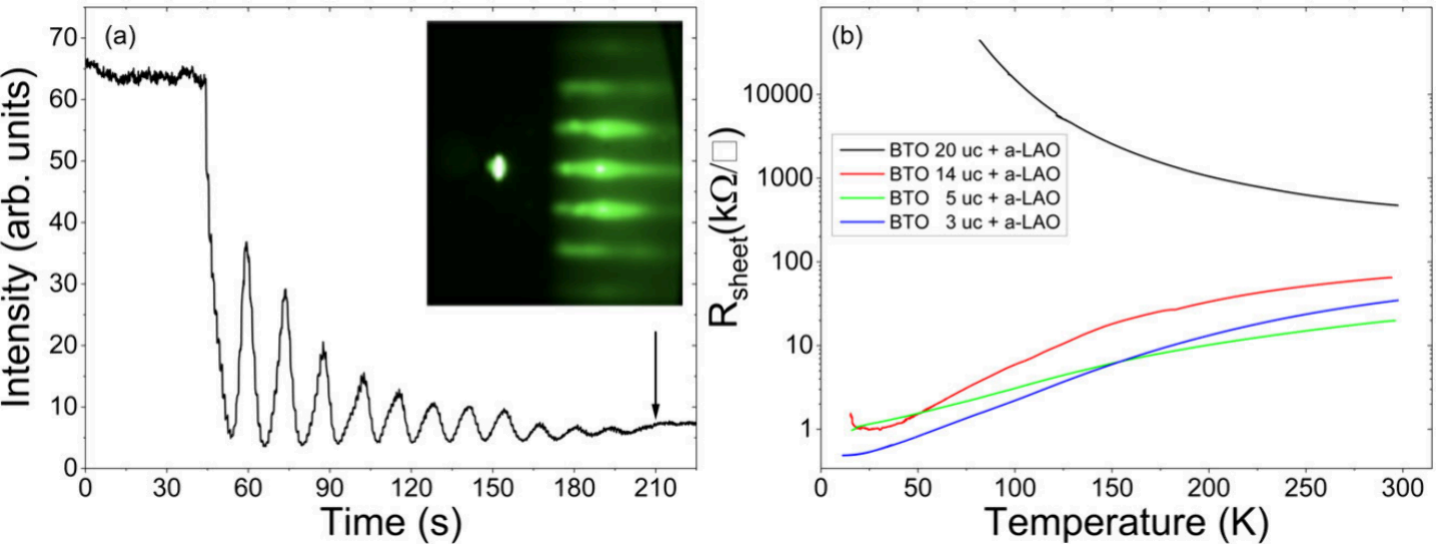
(a) RHEED specular spot intensity oscillations for 12 uc BTO (black arrow indicates the end of the growth) and the corresponding RHEED final pattern. In the selected window of experimental parameters, BTO shows a layer-by-layer growth, with clear dumped oscillations and a final pattern corresponding to a slightly rough but still 2D surface. (b) Temperature-dependent sheet resistance (*R*–*T*) of a set of a-LAO/BTO/STO samples with BTO thickness of 3 uc (blue), 5 uc (green), 14 uc (red), and 20 uc (black) and fixed LAO thickness (2.5 nm). For a fixed a-LAO thickness, samples display metallic-to-insulator behavior by increasing the BTO thickness, up to 14 uc.

SHG measurements at room temperature were employed to assess the polar nature of the system. By varying the polarization angle and utilizing a 45° reflection geometry, SHG allowed us to probe the presence of an in-plane and out-of-plane electrical dipoles due to lattice distortion.^21,22^ To single out the contribution to the SHG signal of the BTO layer, we compare the SHG signals from a-LAO/BTO/STO and a-LAO/STO/STO heterostructures grown in identical experimental conditions and with the same thickness of each corresponding layer. Amorphous-LAO/crystalline-STO (a-LAO/c-LAO) interface exhibits a remarkably high second-harmonic generation (SHG) signal, comparable to that of c-LAO/c-STO interfaces, which origin is attributed to an interfacial electric field, due to a significant number of interfacial oxygen vacancies created during the growth of the amorphous LAO layer.^22^ With the same growth conditions, we expected the oxygen vacancy density and therefore this kind of SHG signal contribution to be very similar in a-LAO/BTO/STO and a-LAO/BTO/STO samples. The difference in the SHG response between them, shown in Figure 2, cannot be explained without invoking an additional contribution, originating from the polar arrangement in the BTO layer, as discussed in the following. Figure 2 shows the comparison of SHG signal as a function of input optical polarization angle α in P-out (Figure 2a) and S-out (Figure 2b) configuration for nominally ferroelectric a-LAO/BTO/STO (red dots) and paraelectric a-LAO/STO/STO (blue dots) heterostructures, for *n* = 14 uc, corresponding to an a-LAO/BTO/STO sample with metallic behavior, as shown in Figure 1b. Comparative analysis of SHG signals from the two heterostructures revealed that BTO-based heterostructures exhibit significantly stronger SHG signals, indicative of pronounced polar distortions. Solid lines represent the best-fit curves for an ideal cubic lattice case with inversion symmetry breaking along the normal direction. This result clearly indicates that the symmetry group is unchanged, and therefore, the in-plane components of the polarization must be vanishing. This behavior is consistent with the expected *c*-axis elongation in BTO films under compressive strain from the STO (001) substrate. To further rule out the possibility that the SHG signal originates from a spurious nonferroelectric contribution, we measured the SHG response in a-LAO/BTO­(*n*)/STO samples with *n* = 4, 5, 6, 7, 10, and 15 uc, keeping the a-LAO thickness fixed at 2,5 nm. As shown in Figure 2c, the SHG intensity increases linearly with BTO thickness, and the best fit suggests a negligible signal in the absence of BTO (*n* = 0). Although surface oxygen vacancies may also contribute to the overall SHG response, their influence is expected to diminish with increasing BTO thickness. Therefore, the observed SHG dependence on the BTO thickness can be attributed to intrinsic polar displacements.

**Figure 2:**
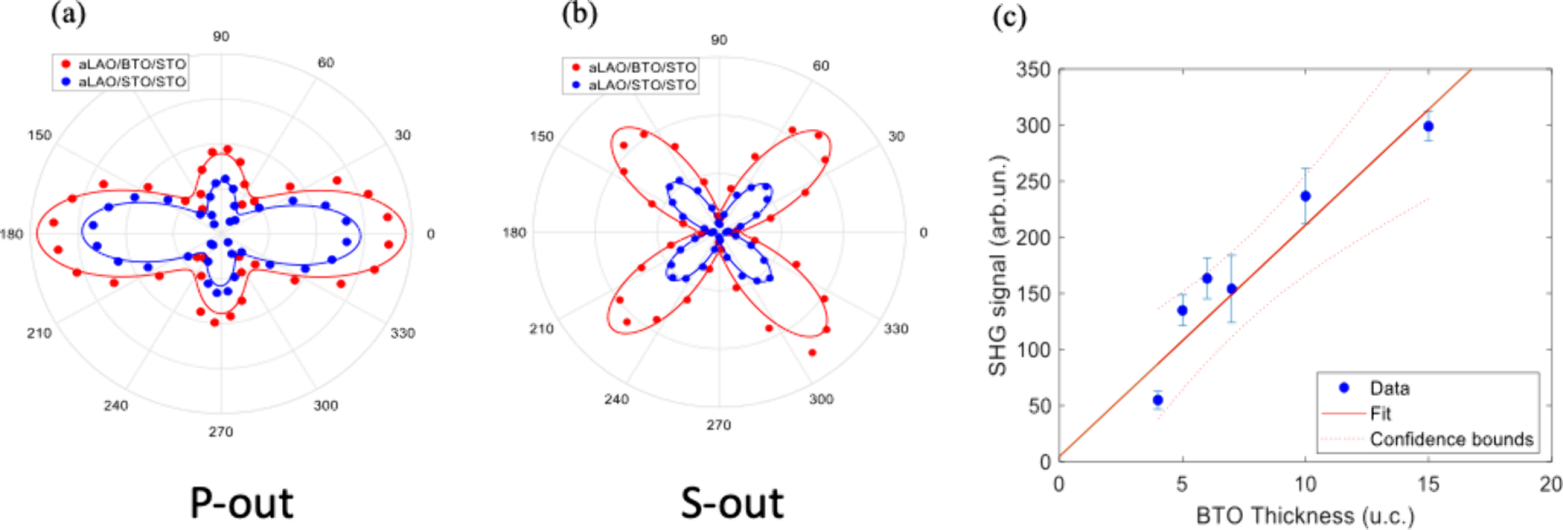
(a and b): SHG intensity vs input optical polarization angle α for a-LAO/BTO­(*n*)/STO (red dots) and a-LAO/STO­(*n*)/STO (blue dots) at an incidence angle of 45°, for *n* = 14 uc. “P-out” label in panel (a) and “S-out” label in panel (b) correspond to optical polarization in the incidence plane (P, corresponding to a 0° angle in the polar plot) and in the sample surface plane (S, corresponding to a 90° angle in the polar plot). It is seen that the SHG has relative maxima in the three directions P-in/P-out (0°–0°), S-in/P-out (90°–0°), and D-in/S-out (45°–90°), where the label “D” indicates the “diagonal” direction in between P and S. The SHG signal is much larger than the one measured on a STO substrate (about 1 order of magnitude; data not shown) and shows the same symmetry in both samples. The best-fit curves (red and blue solid lines in the figure) correspond to those expected for a cubic cell with inversion symmetry breaking along the out-of-plane direction. This result clearly indicates that the symmetry group is unchanged and therefore the in-plane components of the polarization must be vanishing. (c) SHG signal versus BTO thickness, in a-LO/BTO­(*n*)/STO for *n* = 4, 5, 6, 7, 10, 15 uc and a-LAO = 2.5 nm. The blue points are the experimental data, and the error bars represent one standard deviation, while the red line is the corresponding linear fit.

X-ray absorption spectroscopy (at 10 K) provided additional insights into the electronic structure. The persistence of polar displacements at low temperature is proved by X-ray linear dichroism (XLD) measurements, supporting the presence of polar order, even in the sample with the thinnest (3 uc) BTO layer (Figure 3a, lower panel). XAS spectra (Figure 3a, upper panel) differ notably from the XAS typical of the STO-like, purely 3d^0^, Ti^4+^ absorption by the presence of characteristic features, near the L_2,3_ e_g_-peak, that are related to the unusual, non-Jahn–Teller type tetragonal distortion of noncubic and noncentrosymmetric BTO as reported in ref 23.

**Figure 3:**
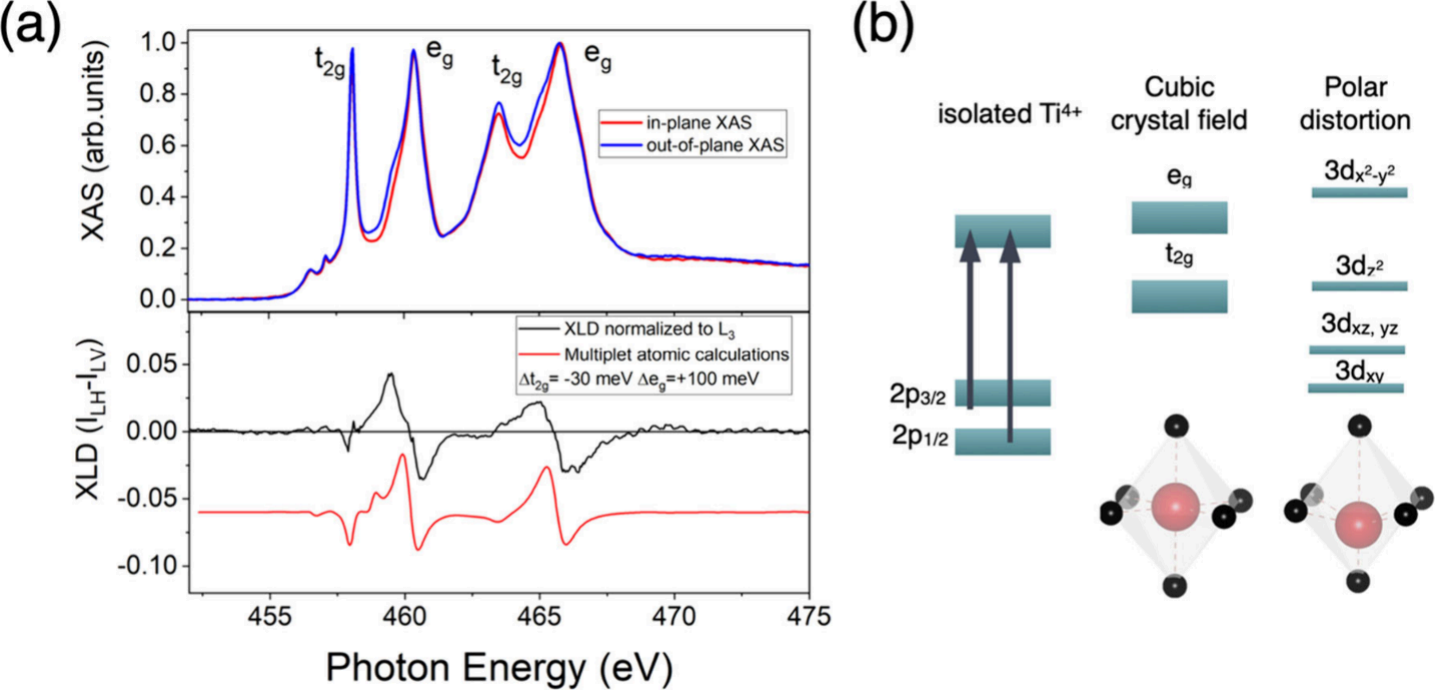
(a) XAS (upper panel) and XLD (lower panel) of a-LAO/BTO­(3uc)/STO; (b) sketch of the orbital crystal splitting in the presence of a polar distortion.

The XLD data, here defined as the difference between the XAS spectra acquired with out-of-plane and in-plane X-ray polarizations, are shown in Figure 3a (bottom panel). The data show a very pronounced dichroism, which is completely different from the one typically measured in the LAO/STO 2DEG.^24,25^

Indeed, XLD in the STO-based system has been shown to be sensitive to the polar displacements of Ti–O ions. In LAO/STO this displacement is due to the presence of a strong confining potential developing when electrons are transferred to the interface and giving rise to t_2g_ orbital splitting due to a polar displacement of the Ti-ions respect oxygen ions in the octahedra, with Ti ions moving toward the STO bulk and the oxygen ions toward the interface. In the case of a ferroelectric material, such as BaTiO_3_, Ti ions are expected to be characterized by a further displacement from the center of the octahedra. In noncentrosymmetric BTO at room temperature, a distinct XLD emerges, as the t_2g_ states are almost degenerate and the e_g_ states show a splitting. This, as evidence of polar distortion, has been associated with the FE state in BTO.^28^ Figure 3 (bottom panel) shows similar results in XLD on our a-LAO/BTO/STO films. In particular, by using multiplet cluster calculations based on the CTM4XAS code,^26^ we find that while the splitting of the t_2g_ conducting bands gives rise to 3d_*xy*_ bands slightly lower in energy than 3d_*xz*_ and 3d_*yz*_, as in the case of epitaxial LAO/STO,^27^ but with a smaller splitting of about 30 meV (see, for example, ref 25), the e_g_ orbitals show opposite splitting. A detailed comparison between centrosymmetric and noncentosymmetric BTO XLD signal is available in the Supporting Information (Figure S2).

Indeed, due to compressive strain, the 3d_*z*_^2^ state is lower in energy than the 3d_*x* ^2^–*y* ^2^_ state, as sketched in Figure 3b. The strong similarity between our XLD and XAS data and those in ref 28, measured on 20 uc ferroelectric BTO films and interpreted as anomalous orbital ordering due to the competition between strain and polarization of BTO, suggests the presence of a strong polar displacement in our 3 uc BTO, as in FE BTO samples.

To validate the metallic nature of the system, we performed ResPES measurements to identify signatures of 2DEG in the Ti-3d states. The valence band map of a-LAO/BTO­(3 uc)/STO exhibited resonant enhancement at the Fermi level, confirming 2DEG formation, with features at −3.5 eV indicative of Ti-3d and O-2p hybridization (Figure 4a). A control BTO/STO sample measured under the same conditions showed no states at the Fermi level. Constant intermediate state (CIS) spectra further confirm that the conduction band is due to partially occupied Ti-3d states, with the characteristic signature of Ti^3+^ 3d^1^ electrons. Deviations in the O-2p CIS spectra, marked by an additional shoulder around 459 eV, are plausibly connected with the ferroelectric-induced distortions in BTO X-ray absorption (Figure 4b), as shown also in the case of thicker BTO films by using XAS.

**Figure 4:**
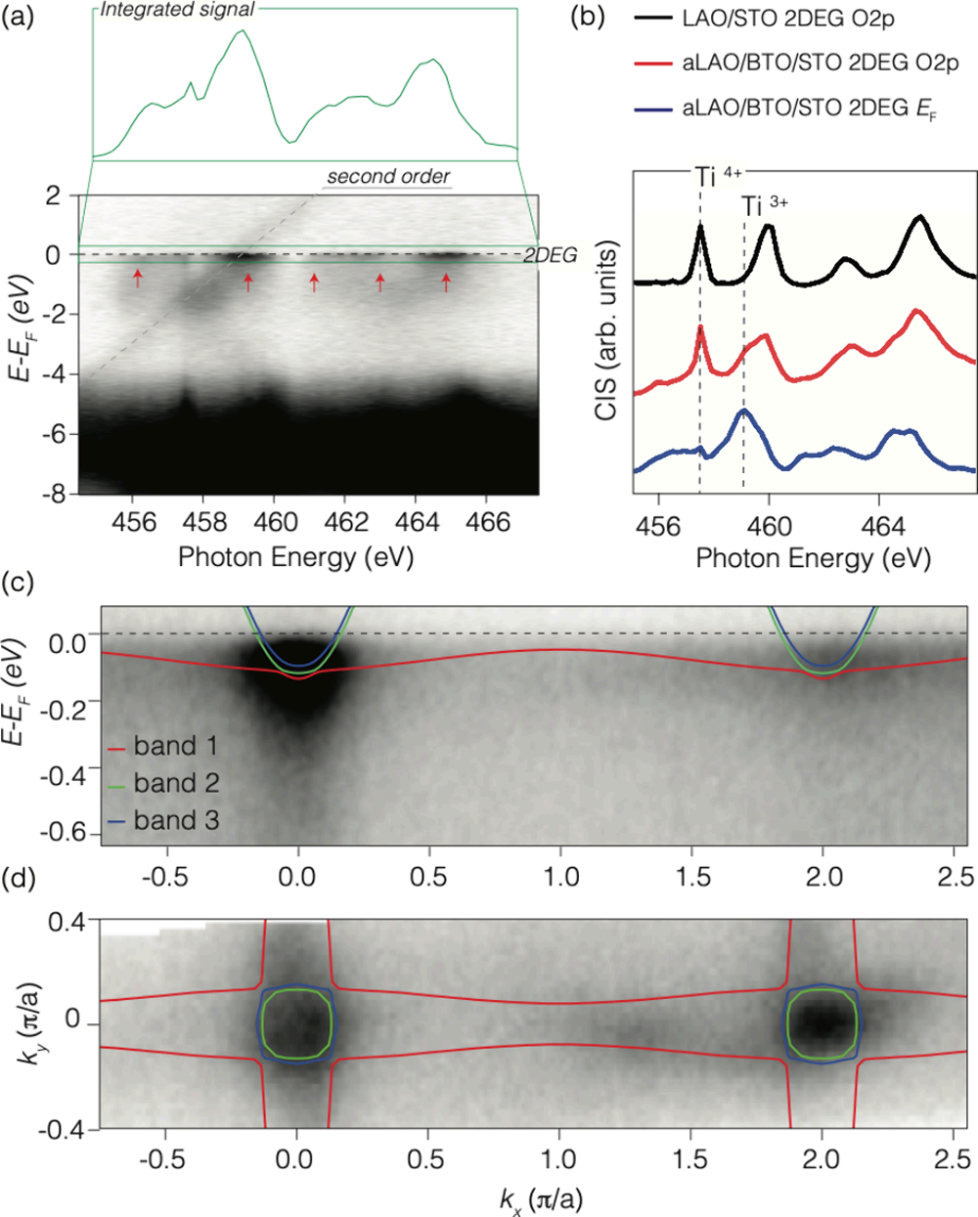
(a) ResPES VB map as a function of the incoming photon energy on a-LAO/BTO­(3uc)/STO 2DEG. (b) CIS data of O 2p bands of LAO/STO (black), O 2p bands (red) and Fermi energy (blue) of a-LAO/BTO­(3uc)/STO. (c) dispersion map along the *kx*-direction in LV polarization with photon energy of 460.2 eV. Continuous lines are the fit using the model Hamiltonian (see the Supporting Information) (red lines, band 1; green line, band 2; blue line, band 3). (d) Fermi surface map obtained from angle resolved ResPES using C+ polarization and a photon energy of 460.2 eV, together with tight binding calculations.

Fermi surface and band dispersion mapping, complemented by tight-binding calculations, reveal a Fermi surface with light and heavy bands of significantly different effective masses (*m*_l_ = 0.56 *m*_e_, *m*_h_ = 14.6 *m*_e_). The carrier density calculated from the soft-ARPES data from the Luttinger count using the tight binding calculations reproducing the data gives about 4 × 10^12^ cm^–2^ for the 3d_*xy*_ bands (about 2 × 10^12^ cm^–2^ for the inner and outer circles, respectively) and about 3 × 10^13^ for the 3d_*xz*,*yz*_ bands. From the Hall effect data as a function of the temperature from 300 K to 2 K (shown in the Supporting Information), we get a carrier density of ∼3 × 10^13^ cm^–2^ at 10 K, the temperature at which the RESPES experiment has been performed. This value of the carrier density is above the Lifshitz transition of standard LAO/STO, at which both 3d_*xy*_ and 3d_*xz*,*yz*_ bands contribute to the transport and is in reasonable agreement with the value obtained from RESPES data. Moreover, the band dispersions are remarkably similar to the BTO surface state reported in refs 29 and 30 . These results show that the 2DEG primarily forms between the BTO layer and the STO substrate.

In summary, we provide evidence of a two-dimensional polar-metal in the a-LAO/BTO/STO heterostructure, and we demonstrate that the 2DEG probed at the a-LAO/BTO interface extends across the BTO film into the STO interfacial unit cells. RESPES data show indeed that the 2DEG is present in the BTO. However, at least in the case of a very thin BTO layer as the one measured by RESPES in the manuscript, it is unlikely that the 2DEG is confined in the BTO alone, as in standard LAO/STO the thickness of the 2DEG at low temperature is between 5 and 10 nm. BTO shows strong polar distortions, compatible with a FE-BTO stabilized by compressive strain and low electron doping. This novel phase aligns with theoretical predictions for strain-engineered polar metals and could pave the way for exploring switchable polarity in oxide 2DEGs. The integration of a ferroelectric metal in stoichiometric BTO, with its enhanced polarization and transition temperature, represents a significant advancement compared with doped STO-based systems. The potential application of polar metals as electrodes to address critical thickness limitations in ferroelectric nanocapacitors^31^ presents promising future directions, making this heterostructure a notable advancement in the field of oxide 2DEG engineering.

## Methods

For the PLD growth, an excimer laser with a wavelength of 248 nm, pulse duration of 25 ns, fluence of 1.5 J/cm^2^, and repetition rate of 1 Hz was employed to deposit films onto (001)-oriented TiO_2_-terminated SrTiO_3_ (STO) substrates. BTO films were grown at 730 °C under an oxygen partial pressure of 10^–1^ mbar, while a-LAO films were deposited at room temperature under a pressure of 10^–4^ mbar. For a-LAO, a minimum thickness of 2.5 nm is required for conductivity, as samples with thinner a-LAO layers are insulating.

## Supplementary Material



## Notes

Plot of the Hall measurement versus temperature of the a-LAO/BTO­(3uc)/STO heterostructure; description of the technical aspect of SHG, ResPES and CIS measurements; details regarding XAS and XLD simulations and of tight binding calculations (PDF)

## ∇

For Deepak Kumar: Maryland Quantum Materials Center and Department of Physics, University of Maryland, College Park, MD 20742, USA
