# Supplementary material for: Signatures of Polar Metal Phase in the Quasi-2D Electron System in PLD-Grown Amorphous-Epitaxial Oxide Heterostructures
Source: Nano Lett. 2025 Aug 31;25(36):13608–13. doi: 10.1021/acs.nanolett.5c03409 (PMC12426986; doi:10.1021/acs.nanolett.5c03409)
Supplement: Supplementary file 1 [file nl5c03409_si_001.pdf]

Signatures of polar metal phase in the quasi-2D electron system in PLD-grown amorphous-epitaxial oxide heterostructures

Alessia Sambri[1*], Yu Chen[1], Federico Mazzola[1], Emiliano Di Gennaro[2], Andrea Rubano[2], Martando Rath[1], Domenico Paparo [2,3], Marco Caputo[4], Alla Chikina[4], Deepak Kumar[1§], Vladimir N. Strocov[4], Marco Salluzzo[1], Fabio Miletto Granozio[1].

1 *CNR-SPIN, Institute for SuPerconductors, INnovative materials and devices, Unit of Naples, Complesso Universitario di Monte Sant'Angelo, via Cinthia, 80126, Napoli, Italy.*
2 *Dipartimento di Fisica "E. Pancini", Università degli Studi di Napoli "Federico II", Complesso Universitario di Monte Sant'Angelo, via Cinthia, 80126 Napoli, Italy.*
3 *CNR-ISASI, Institute of Applied Sciences and Intelligent Systems "E. Caianiello", 80078 Pozzuoli, NA, Italy.*
4 *Swiss Light Source, Paul Scherrer Institut, CH-5232 Villigen PSI, Switzerland.*

§now at *Maryland Quantum Materials Center and Department of Physics, University of Maryland, College Park, Maryland 20742, USA.*

Transport measurements

The observed conductivity in the a-LAO/BTO/STO heterostructure is attributed to the formation of oxygen vacancies via redox reactions, particularly induced by the high oxygen affinity of Al atoms from the LAO plasma plume. This mechanism, widely recognized, has been leveraged to create two-dimensional electron gases (2DEGs) in systems such as Al/STO, Al/TiO_2, and Al/BTO through Molecular Beam Epitaxy, as well as in a range of amorphous $LaAlO_3$ and other conducting oxide interfaces grown by PLD. Hall measurements on a-LAO/BTO(3uc)/STO are shown in Figure S1 for different temperature. The corresponding temperature dependence of carrier density is shown in the inset.

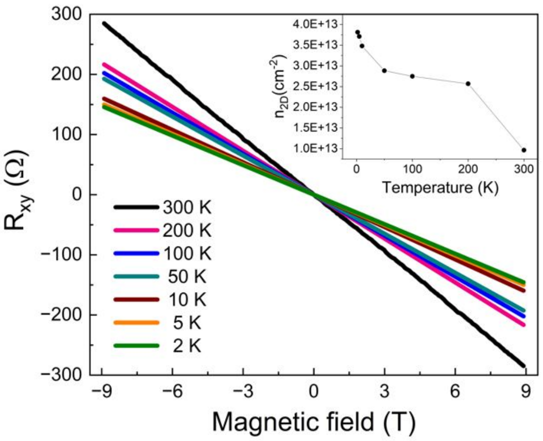

S1: Hall measurements on a-LAO/BTO(3uc)/STO for different temperatures and corresponding temperature dependence of carrier density n_{2D}.

SHG measurements

To investigate polar distortion linked to ferroelectricity, we utilized optical Second Harmonic Generation (SHG), as conventional Piezoelectric Force Microscopy (PFM) cannot be applied to BTO films embedded in a-LAO/BTO/STO heterostructures. SHG is ideal for probing polar order because it detects the frequency doubling of light waves, which occurs only in materials lacking inversion symmetry. The SHG emission depends on the second-order susceptibility tensor $\chi(2)$, with nonvanishing components determined by the

material's symmetry. In inversion-symmetric materials, all $\chi(2)$ components vanish, but surfaces or interfaces naturally break this symmetry along the normal to the sample surface. Consequently, if ferroelectric (FE) polarization is along this axis, the material's symmetry group remains unchanged, though a significant increase in SHG intensity is expected due to the larger nonlinear tensor components typical of FE materials. If the polarization has in-plane components, additional tensor components can be detected via SHG anisotropy measurements.

ResPES and CIS measurements

Measurements were performed at the soft-X-ray ARPES endstation at the ADRESS beamline of the Swiss Light Source (SLS) [S1,S2]. The x-ray absorption spectroscopy (XAS) data were measured in the total electron yield (TEY) mode. ResPES valence band (VB) spectra were acquired as function of x-ray photon energy tuned between 450 and 468 eV, i.e. the energy range for the resonant absorption at the Ti L-edge. From ResPES measurement, we obtained constant intermediate state (CIS) spectra related to the valence TiO 2*p*-band region and the Fermi-level region, by integrating the ResPES map in the given relevant energy window (between -8 and -4 eV for O 2*p* CIS, and between -0.5 and E_F for the Fermi level CIS).

Details of the XAS and XLD simulations

Atomic multiplet scattering simulations including charge transfer (CT) effects were done using charge transfer ligand field multiplet theory, implemented in the CTM4XAS software [S3]. Atomic multiplet simulations in Fig. 2 for Ti^{4+} ions were performed in D4h symmetry and included a charge transfer (CT) term to account for the hybridization between Ti-3*d* and O-2*p* states in the TiO_6 cluster, i.e. a $3d^0+3d^1\underline{L}$ configuration, where $\underline{L}$ indicates a hole in the O-2*p* band. The CT is included in an Anderson impurity type of model where two parameters are necessary to describe the system: the energy difference between the Ti-*3d* and the ligand band (called Δ here), and the difference U_{pd}-$\underline{U}_{dd}$ between the core hole potential (U_{pd}) and the Hubbard U value (U_{dd}) for Ti-d electrons. Here we used, Δ=3.5 eV and U_{pd}-$\underline{U}_{dd}$ = 1.5eV. Finally, the transfer integrals used were 2 and 1 for b_1, a_1 (d_{x2-y2}, d_{z2}) and b_2, e (d_{xy}, d_{xz}/d_{yz}) orbitals, respectively. The Slater Integrals were reduced by 80% respect the corrected Hartree-Fock values. The broadening used were 0.1 eV for the Gaussian broadening, to account for the energy resolution, and different Lorentzian broadening for each of the 4 peaks of the spectrum, namely half width at half maximum of 0.15 eV, 0.45 eV, 0.65 eV, and 0.6 eV for the four XAS peaks (from the first to the last). In the calculations of the Ti-3*d* XLD we changed only the crystal field parameters. In particular, good agreement between the shape and the amplitude of the XLD spectra and calculations is obtained using Dt= 0.012 eV and Ds=0.010 eV, corresponding to Δ t_{2g}= -30 meV and Δ e_g=100 meV. Thus, the ordering of in-plane and out of plane orbitals is opposite for t_{2g} and e_g states, which happens only in the case of non-centrosymmetric polar distortions. We also performed calculations assuming on the other hand just a centrosymmetric tetragonal distortion. In this case the e_g and t_{2g} splitting is expected to have the same sign, with an e_g splitting roughly two times the t_{2g} one, i.e. Dt= 0.021429 eV and Ds= 0.002857 eV, corresponding to Δ t_{2g}=50 meV and Δ e_g=100 meV. In this case the XLD spectra at the energies corresponding to t_{2g} features is notably different from the experimental data.

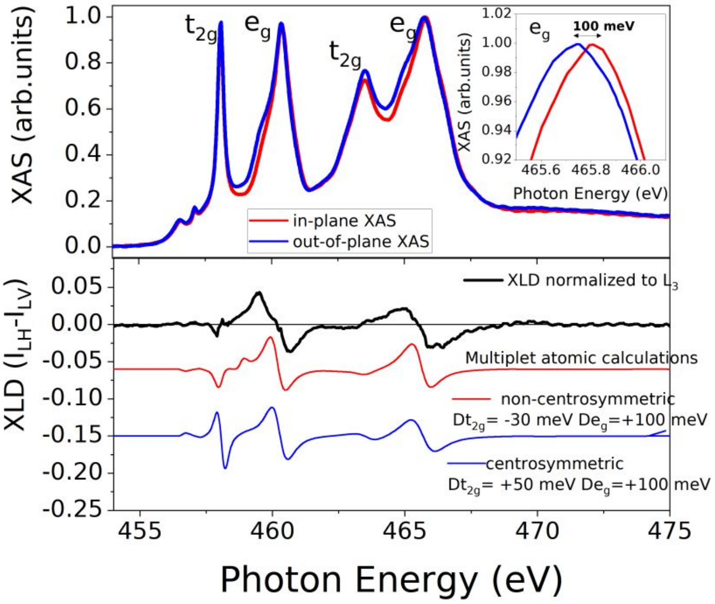

Figure S2: *XAS (upper panel) and XLD (lower panel) of a-LAO/BTO(3uc)/STO. The inset of the upper panel is a zoom showing the e_g orbitals splitting. In the lower panel, the black line shows the XLD signal normalized to L_3, while the red and blue lines represent the simulation in case of non-centrosymmetric and centrosymmetric structural distorsion, respectively.*

Tight binding band calculations

For the tight binding calculation, we consider a typical three bands model Hamiltonian as follows:

$$H = \begin{pmatrix} \epsilon_{xy} & 0 & 0 & 0 & \lambda & -i\lambda \\ 0 & \epsilon_{yz} & i\lambda & -\lambda & 0 & 0 \\ 0 & -i\lambda & \epsilon_{zx} & i\lambda & 0 & 0 \\ 0 & -\lambda & -i\lambda & \epsilon_{xy} & 0 & 0 \\ \lambda & 0 & 0 & 0 & \epsilon_{yz} & -i\lambda \\ i\lambda & 0 & 0 & 0 & i\lambda & \epsilon_{zx} \end{pmatrix}$$

$\lambda = 0\ eV\ or\ \lambda = \frac{1}{120} eV$ in case of three or six bands. λ is a related to the SOC coupling term, and is either zero (as in the calculations shown in Figure 4) or taken equal $\lambda = \frac{1}{120} eV$which are typical values for the LAO/STO 2DEG [S4]

Here

$\epsilon_{xy} = 2t_l[2 - \cos(k_x a) - \cos(k_y a)] + o_1$

$\epsilon_{yz} = 2t_l[1 - \cos(k_y a)] + 2t_h[1 - \cos(k_x a)] + o_2$

$\epsilon_{zx} = 2t_h[1 - \cos(k_y a)] + 2t_l[1 - \cos(k_x a)] + o_2$

and o_1,o_2 are the band bottoms of light and heavy bands in the case $\lambda = 0$.

SI References

SI 1) V.N. Strocov, X Wang, M. Shi, M. Kobayashi, J. Krempasky, C. Hess, T. Schmitt, L. Patthey, Soft-X-ray ARPES facility at the ADRESS beamline of the SLS: concepts, technical realisation and scientific applications, J. Synchrotron Rad. **21** (**2014**) 32 https://doi.org/10.1107/S1600577513019085

SI 2) V.N.Strocov, T. Schmitt, U. Flechsig, T. Schmidt, A. Imhof, Q. Chen, J. Raabe, R. Betemps, D. Zimoch, J. Krempasky, X. Wang, M. Grioni, A. Piazzalunga, L. Patthey, High-resolution soft X-ray beamline ADRESS at the Swiss Light Source for resonant inelastic X-ray scattering and angle-resolved photoelectron spectroscopies, J. Synchrotron Rad. 17, (**2010**) 5 10.1107/S0909049510019862

SI 3) Stavitski, E.; de Groot, F. M. F. The CTM4XAS Program for EELS and XAS Spectral Shape Analysis of Transition Metal L Edges. Micron **2010**, 41, 687.

SI 4) Vaz D., Noël P., Johansson A., Göbel B., Bruno F., Singh G., McKeown-Walker S.,Trier F., Vicente-Arche L., Sander A., Valencia S., Bruneel P., Vivek M., Gabay M., Bergeal N., Baumberger F., Okuno H., Barthélémy A., Fert A., Vila L., Mertig I., Attané J., Bibes M., Mapping spin–charge conversion to the band structure in a topological oxide two-dimensional electron gas, Nature Materials 18, 1187-1193 (**2019**).
